# Promoter/enhancer-based controllability of regulatory networks

**DOI:** 10.1038/s41598-022-07035-4

**Published:** 2022-03-03

**Authors:** Prajwal Devkota, Stefan Wuchty

**Affiliations:** 1 Department of Computer Science, University of Miami, Miami, FL 33146 USA; 2 Department of Biology, University of Miami, Miami, FL 33146 USA; 3 Sylvester Comprehensive Cancer Center, Univ. of Miami, Miami, FL 33136 USA; 4 Present Address: Scipher Medicine Inc, Waltham, MA 02453 USA

**Keywords:** Computational biology and bioinformatics, Gene regulatory networks

## Abstract

Understanding the mechanisms of tissue-specific transcriptional regulation is crucial as mis-regulation can cause a broad range of diseases. Here, we investigated transcription factors (TF) that are indispensable for the topological control of tissue specific and cell-type specific regulatory networks as a function of their binding to regulatory elements on promoters and enhancers of corresponding target genes. In particular, we found that promoter-binding TFs that were indispensable for regulatory network control regulate genes that are tissue-specifically expressed and overexpressed in corresponding cancer types. In turn, indispensable, enhancer-binding TFs were enriched with disease and signaling genes as they control an increasing number of cell-type specific regulatory networks. Their target genes were cell-type specific for blood and immune-related cell-types and over-expressed in blood-related cancers. Notably, target genes of indispensable enhancer-binding TFs in cell-type specific regulatory networks were enriched with cancer drug targets, while target genes of indispensable promoter-binding TFs were bona-fide targets of cancer drugs in corresponding tissues. Our results emphasize the significant role control analysis of regulatory networks plays in our understanding of transcriptional regulation, demonstrating potential therapeutic implications in tissue-specific drug discovery research.

## Introduction

Transcriptional mechanisms allow cells to respond to signals or perform their functions by regulating gene expression, a vital process in all organisms that is orchestrated by transcription factors (TF). Gene regulation is usually controlled through combinations of TFs, allowing a comparatively small set of TFs to govern and maintain complex cell-specific states^1^. Using regulatory elements to control gene expression TFs bind such elements in the promoter region near the transcription start site of a gene, while others recognize such elements on distal enhancer regions.

While TFs can bind their cognate sites in a context dependent fashion, indicating that the same factor could bind to either promoter and/or enhancer regions, the functions of such regulatory regions as well as the combination of TFs that exert control in different tissue, cell-types and cell lines are not well understood. Furthermore, recent studies have also highlighted links between disease-associated variants in promoter/enhancer based regulatory DNA and breast cancer^2^, prostate cancer^3^, colorectal cancer^4^, renal cancer^5^, lung cancer^6^, and melanoma^7,8^. As a consequence, the identification of key TFs that exert control through their regulatory elements in promoter and enhancers in tissue-specific networks can lead to further understanding of the complex ways of gene regulation, potentially indicating to points of therapeutic intervention.

To understand the roles played by different TFs through their regulatory elements in maintaining the cell state, we determined the TFs that exert topological control over the tissue and cell-type specific networks through binding promoters and enhancers. Previous studies indicated that control proteins in protein–protein interaction networks are enriched with disease genes and drug targets^9,10^. While such results were obtained from networks that ignored the presence of their interactions in different tissues, regulatory networks are indeed tissue and cell-type specific^11^. As a corollary, we surmised that the disruption of regulation by such control TFs might result in diseases associated with the underlying tissue, suggesting that their target genes support the transition from disease to healthy states, pointing to potential drug targets.

In our analysis of tissue- and cell-type specific regulatory networks between TFs and target genes, we determined TFs that were indispensable for the topological control of the underlying network as a function of their binding of promoter and enhancers of corresponding target genes. Specifically, we found that promoter-binding TFs are mostly indispensable for the control of underlying tissue-specific regulatory networks, while enhancer-binding TFs were indispensable for the control of cell-type specific networks. Furthermore, such tissue-specific indispensable TFs also regulated genes that are overexpressed in cancer tissues and targets of drugs approved for the treatment of corresponding cancers. While indispensable enhancer-binding TFs seemed to play a minor role in the control of tissue-specific regulatory networks, we found that they were enriched with disease, cancer and signaling genes as they controlled an increasing number of cell-type networks, indicating their biological significance. Furthermore, target genes of such enhancer-binding indispensable TFs in cell-type specific regulatory networks were also cancer-specific for blood and immune-related cells.

## Results

**Figure 1:**
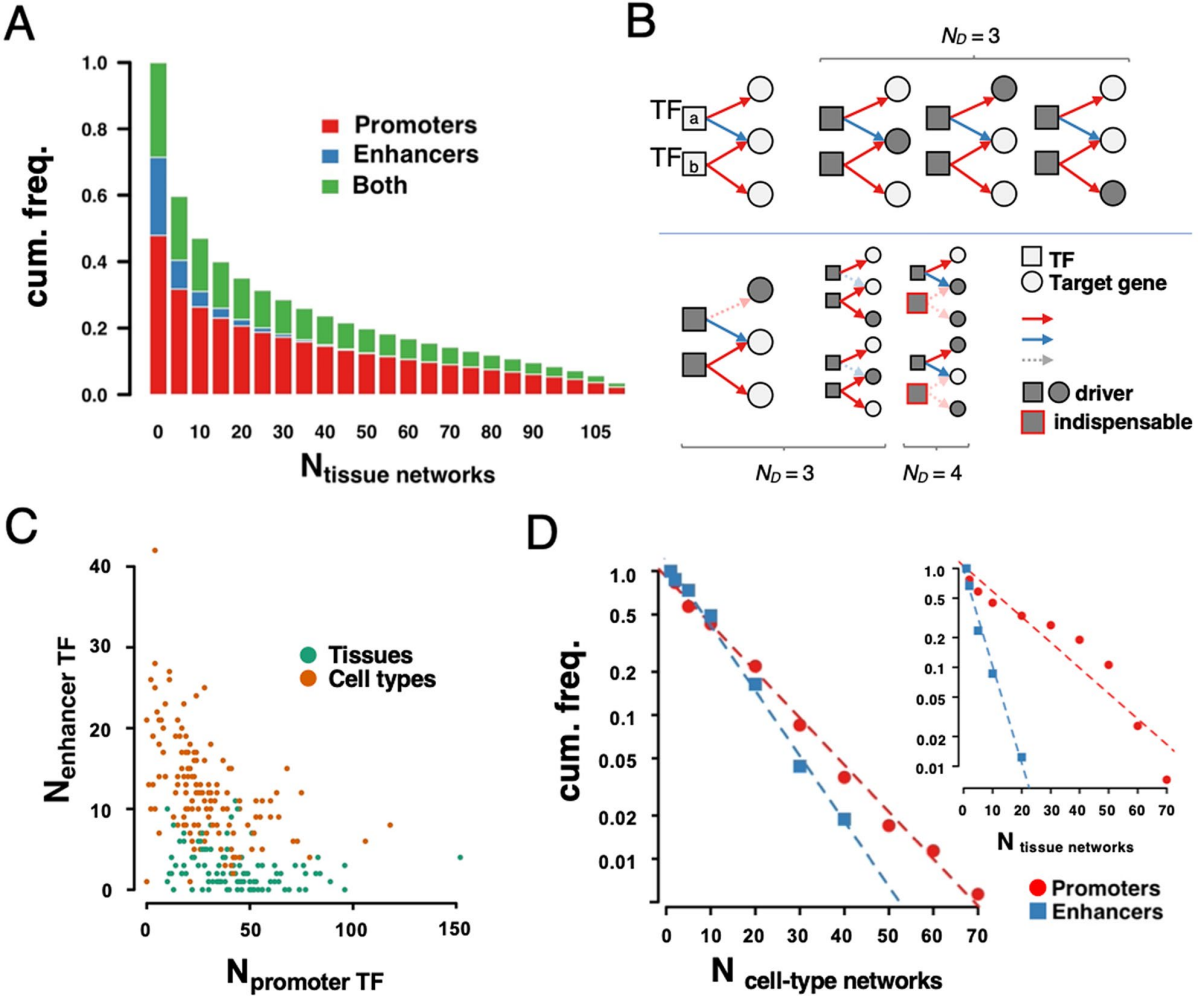
Characteristics of regulatory networks. (**A**) In different tissues, we counted both promoter-and enhancer-binding regulatory interactions between transcription factors (TF) and target genes. Specifically, we observed that promoter-binding regulatory links tend to occur in more tissues while enhancer-binding interactions were more likely to be tissue-specific. (**B**) In the schematic toy model of our controllability framework we considered a regulatory network as a bipartite graph, where TFs and target genes in regulatory interactions represent partitions. Specifically, the application of a maximum matching algorithm allowed us to find three different topological configurations with *N*_*D*_ = 3 driver nodes. To find TFs that are indispensable for the control of the underlying regulatory network, we separately eliminated corresponding promoter/enhancer-binding interactions of a given TF. Determining the number of driver nodes in the network thus obtained, we observed that the elimination of promoter or enhancer-binding interactions of TF_a_ did not change the number of driver nodes. However, the removal of promoter-binding interactions of TF_b_ in our toy model increased the number of driver nodes, suggesting that the promoter-binding TF_b_ is indispensable for the control of the underlying regulatory network. In (**C**), we counted the number of TFs that were indispensable for the control of tissue and cell-type specific regulatory networks as a function of promoter and enhancer binding regulatory interactions. In particular, we significantly found that cell-type specific networks were dominated by TFs that exerted their control through enhancer binding, while we observed the opposite in tissue-specific networks. (**D**) In turn, we counted the number of cell-type and tissue (inset) specific regulatory networks where a given TF was found indispensable for their control, pointing to cumulative frequency distributions that generally followed exponential decays.

We obtained 111 tissue- and 146 cell type specific regulatory networks from^12^, connecting transcription factors to their corresponding target genes (see Material and Methods). Each regulatory interaction was classified as promoter and/or enhancer based, indicating that the corresponding TF occupies binding sites on promoters/enhancers to control their target genes. In Fig. 1A, we counted the appearance of such promoter/enhancer-binding regulatory interactions in different tissues and cell types (Fig. S1). Notably, we observed that regulatory interactions of TFs that bind promoters tend to appear more frequently in both tissues and cell types. Furthermore, we determined the fractions of promoter and enhancer-binding regulatory interactions in tissue and cell-type specific networks. We found that promoter-binding interactions had a significantly higher appearance in tissues compared to cell types, while we observed the opposite when we considered enhancer-binding regulatory links (P < 0.05, Wilcoxon rank-sum test, Fig. S2).

To investigate driver nodes that ensure the structural controllability of linear dynamics^13^ we mapped each regulatory network to a bipartite graph, where heads and tails of directed edges represent the partitions. Specifically, we determined the largest subset of interactions in each bipartite network called a maximum matching, where no two interactions shared a common start and end point. Unmatched nodes in any maximum matching were previously shown to be driver nodes, allowing the structural control of the whole underlying network^13^. To further assess the relevance of TFs we considered the topological consequences of the removal of their regulatory interactions through either promoters or enhancers. In particular, we defined an indispensable promoter-binding transcription factor if the total number of driver nodes increased in a maximum matching after the removal of promoter-binding regulatory links, while we kept enhancer-binding interactions of the given TF. In turn, we considered the presence of an indispensable enhancer-binding transcription factor if the number of driver nodes increased after the removal of all enhancer-binding regulatory links of a TF, while we kept promoter-binding interactions (Fig. 1B). As a corollary, we also determined generally indispensable TFs, when the number of driver nodes increased upon deletion of a TF and its interactions^10^. In each tissue and cell-type specific regulatory network, we determined the number of indispensable promoter- and enhancer-binding TFs. In Fig. 1C, we significantly found that cell-type specific networks are dominated by indispensable enhancer-binding TFs, while tissue specific network are rather controlled by indispensable promoter-binding TFs.

We further counted the number of tissue specific regulatory networks, where we found indispensable promoter/enhancer-binding TFs (Fig. 1D). Our results point to an exponential distribution, suggesting that a minority of TFs appeared to be indispensable for a majority of tissues. Notably, the observed decay is faster when we considered indispensable enhancer-binding TFs, corroborating the initial observation that they appear more frequently than indispensable enhancer-binding TFs in tissue-specific regulatory networks (inset, Fig. 1C). We observed similar results when we considered indispensable promoter/enhancer-binding TFs in cell-type specific networks, that point to comparable decays (Fig. 1C). As a final comparison, we considered generally indispensable TFs and observed that the distribution of the appearance of a given generally indispensable TF decays as a power-law in both tissue and cell-type specific regulatory networks (Fig. S3).

### Tissue networks

**Figure 2:**
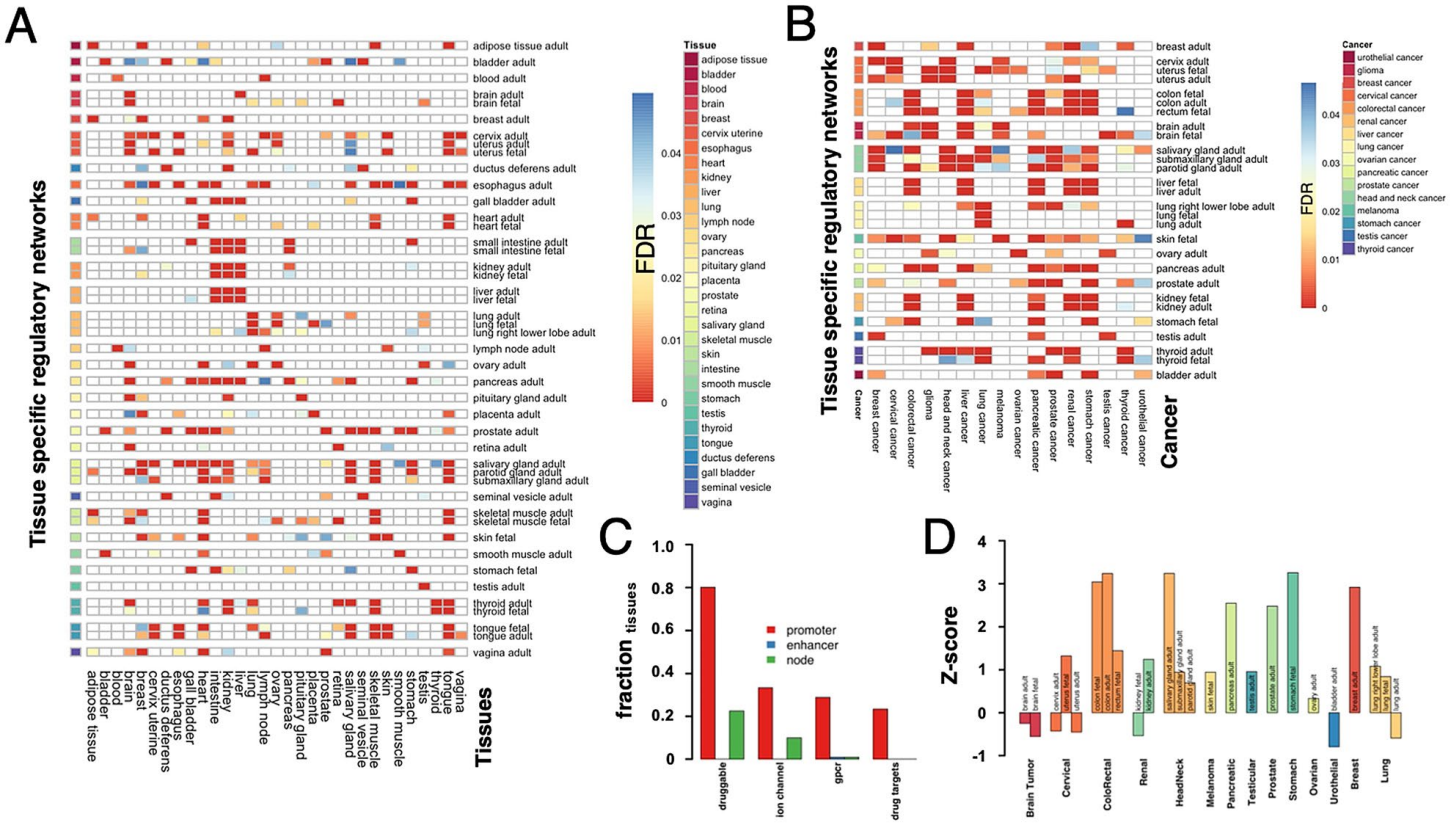
Tissue and cancer specificity of target genes of promoter-binding indispensable transcription factors (TF). (**A**) Investigating target genes that were regulated by indispensable promoter-binding TFs, we determined their enrichment in sets of genes that were significantly expressed in 30 different tissues (FDR < 0.05, Fisher’s exact test). In (**B**), we corroborated our findings by determining the enrichment of cancer-specific genes in 16 different types of cancers in sets of target genes of indispensable promoter-binding TFs. In particular, we observed that cancer-specific genes were indeed enriched in all corresponding tissue-specific regulatory networks. In (**C**), we determined the enrichment of the families of druggable genes, FDA-approved drug-targets, ion channels and G protein-coupled receptors (GPCR) in sets of target genes that were regulated by indispensable promoter/enhancer-binding TFs in each tissue-specific network. (FDR < 0.05, Fisher’s exact test). Generally, we found that target genes of indispensable promoter-binding TFs were enriched with such gene sets in more tissue-specific regulatory networks than target genes of enhancer-binding or generally indispensable TFs. (**D**) Focusing on a list of drugs that were approved for different cancers from the National Cancer Institute (NCI) we determined the enrichment of their drug targets in the sets of target genes of indispensable promoter-binding TFs in tissues closest to corresponding cancer types. Except for Brain Tumor and Urothelial Cancer, we found that drug targets significantly appeared in sets of target genes of indispensable promoter-binding TFs in the corresponding tissue-specific regulatory networks.

Since enhancer-binding regulatory interactions tend to appear in a smaller number of tissues compared to promoter-binding links, we assumed that target genes regulated through indispensable enhancer-binding TFs are likely to be tissue-specific. Consequently, we obtained tissue-specific genes in 31 different tissues from the Human Protein Atlas database^14^, defining that a gene is tissue-specific if its mRNA level is at least four-fold higher than the corresponding average expression level in all other tissues. In Fig. S4, we determined the enrichment of target genes that were regulated by promoter/enhancer based regulatory interactions in the sets of tissue-expressed genes (Fisher’s exact test, FDR < 0.05). Expectedly, we found that target genes regulated through enhancer-binding TFs contained a significantly larger proportion of tissue-specific genes. In turn, such tissue specificity was weak when we considered all target genes regulated by promoter-binding TFs. However, when we considered the target genes of indispensable promoter-binding TFs, we found that genes expressed in these tissues were significantly better enriched (Fig. 2A). When we focused only on regulatory networks relevant to the tissue data from the Human Protein Atlas database^14^ we found that such enrichment was significant in 30 (out of 31) tissues. Most surprisingly, hardly any tissue specificity existed among the target genes of indispensable enhancer-binding TFs (Fig. S5). Furthermore, such results were corroborated as well when we considered generally indispensable transcription factors (Fig. S6).

We corroborated our findings by considering the enrichment of cancer-specific genes of 16 different cancer types that we obtained from the Pathology Atlas^15^. In Fig. 2B, we observed that target genes of indispensable promotor-binding TFs in tissue-specific regulatory networks were significantly enriched with genes that were expressed in corresponding cancer types. Like tissue specificity, target genes of indispensable enhancer-binding TFs were not cancer-specific (Fig. S7). Furthermore, we observed that genes regulated by generally indispensable TFs showed very weak cancer specificity compared to target genes of indispensable promoter-binding TFs (Fig. S8).

**Figure 3:**
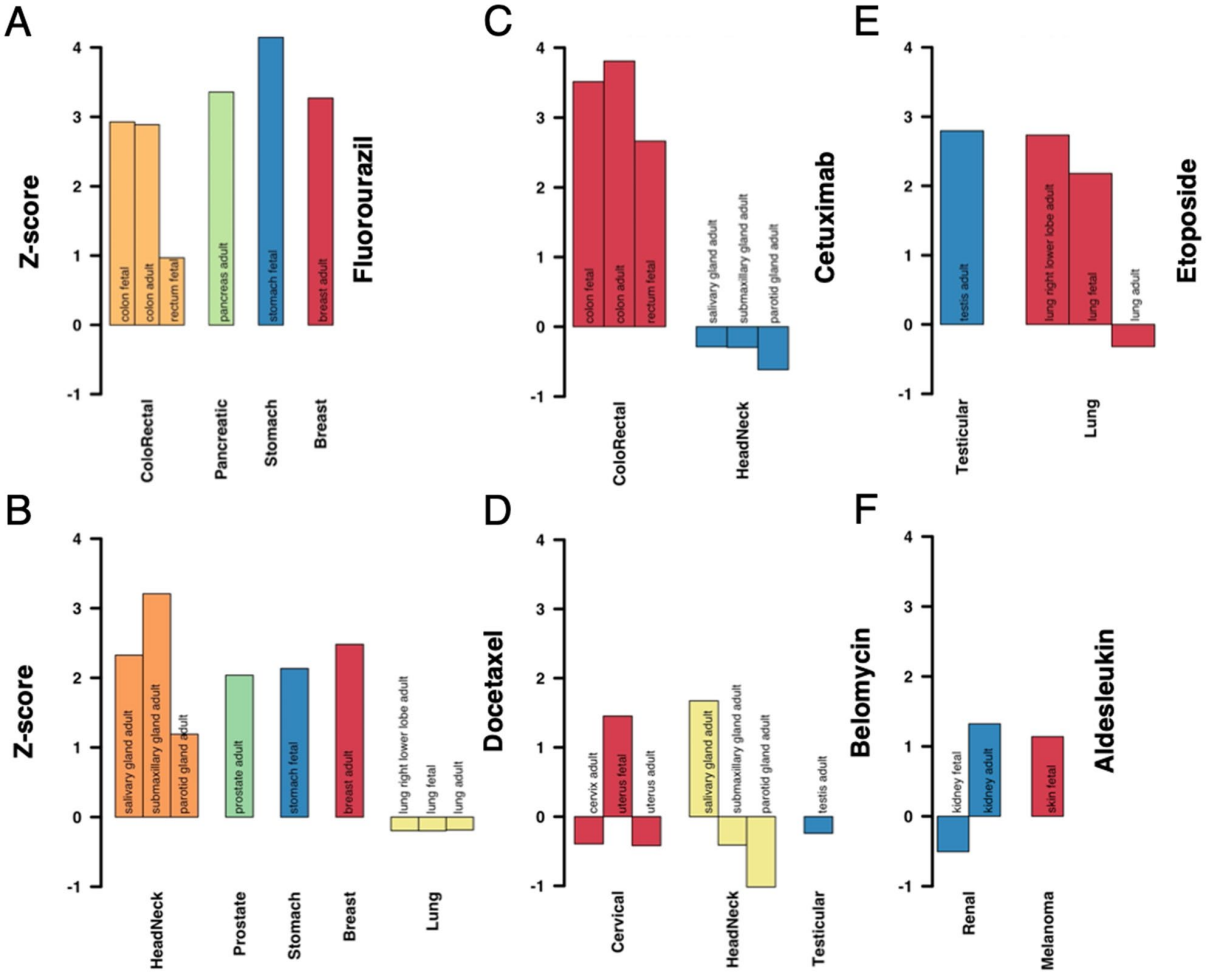
Enrichment of drug targets of individual cancer drugs in sets of target genes of indispensable promoter-binding TFs in disease affected tissues. (**A**) The gene targets of Fluorouracil, a drug approved for colorectal, pancreatic, stomach and breast cancer, were broadly enriched in target sets of indispensable promoter-binding TFs in tissue-related regulatory networks (except fetal rectum tissue in colorectal cancer). (**B**) Drug targets of Docetaxel were enriched in sets of target genes of indispensable promoter-binding TFs in tissues that developed head and neck, prostate, stomach, and breast cancers. Even though this drug was approved for lung cancer, we found a dilution of its drug targets in lung-specific tissues. (**C**) Drug targets of Cetuximab were enriched in sets of target genes of indispensable promoter-binding TFs in colon-specific tissues affected by colorectal cancer. Although Cetuximab is approved for head and neck cancer, we did not find any enrichment signals in gland tissues. (**D**) We found mixed results when we considered drug targets of Bleomycin, a drug that was approved for head and neck, cervical and testicular cancer. (**E**) Etoposide drug targets were enriched in tissues that were associated with testicular cancer and lung cancer. (**F**) Aldesleukin drug targets were weakly enriched in sets of target genes of indispensable promoter-binding TFs in tissues related to renal cancer and melanoma.

To indicate the biological significance of indispensable promoter/enhancer binding TFs in regulatory networks, we hypothesized that they might play a role in the transition between healthy and disease states. Considering genes responsible for the transition from disease to a healthy state, we utilized 1,982 drug targets that were approved by the Food and Drug Administration (FDA)^16^. We also considered a set of 4808 druggable proteins as they carry protein folds, favoring interactions with chemical compounds^17^. Investigating target genes of indispensable promoter-binding transcription factors in each tissue network separately, we found that druggable proteins and drug targets were significantly overrepresented (Fisher’s exact test, P < 0.05, FDR adjusted) in a large fraction of the networks (Fig. 2C). Focusing on the different families of druggable proteins, we found that Ion Channels and G protein-coupled receptors were overrepresented as well. However, we did not observe such enrichment levels of druggable proteins in the target genes of indispensable enhancer-binding or generally indispensable TFs (Fig. 2C). As a corollary, we hypothesized that drug targets of approved cancer drugs were also target genes of indispensable promoter-binding TFs. Considering drug targets of 74 approved drugs for 18 different cancer types from the National Cancer Institute (NCI) through DGIdb^18^, we found that, except for Brain Tumor and Urothelial Cancer, the targets of all the drugs that treat a particular cancer were enriched in target genes of indispensable promoter-binding TFs in the corresponding tissues (Fig. 2D). Surprisingly, such enrichment levels were not observed when we considered target genes of indispensable enhancer-binding and generally indispensable TFs (Fig. S9). Furthermore, we selected six different drugs, Fluorouracil, Docetaxel, Cetuximab, Bleomycin, Etoposide, and Aldesleukin (Fig. 3) that treat multiple cancers and had at least one target in our networks. Determining the enrichment of each drug’s targets in sets of target genes that were regulated through indispensable promoter-binding TFs we found that largely drug targets were enriched in tissue networks corresponding to the cancer type the underlying drug was approved for.

### Cell-type networks

**Figure 4:**
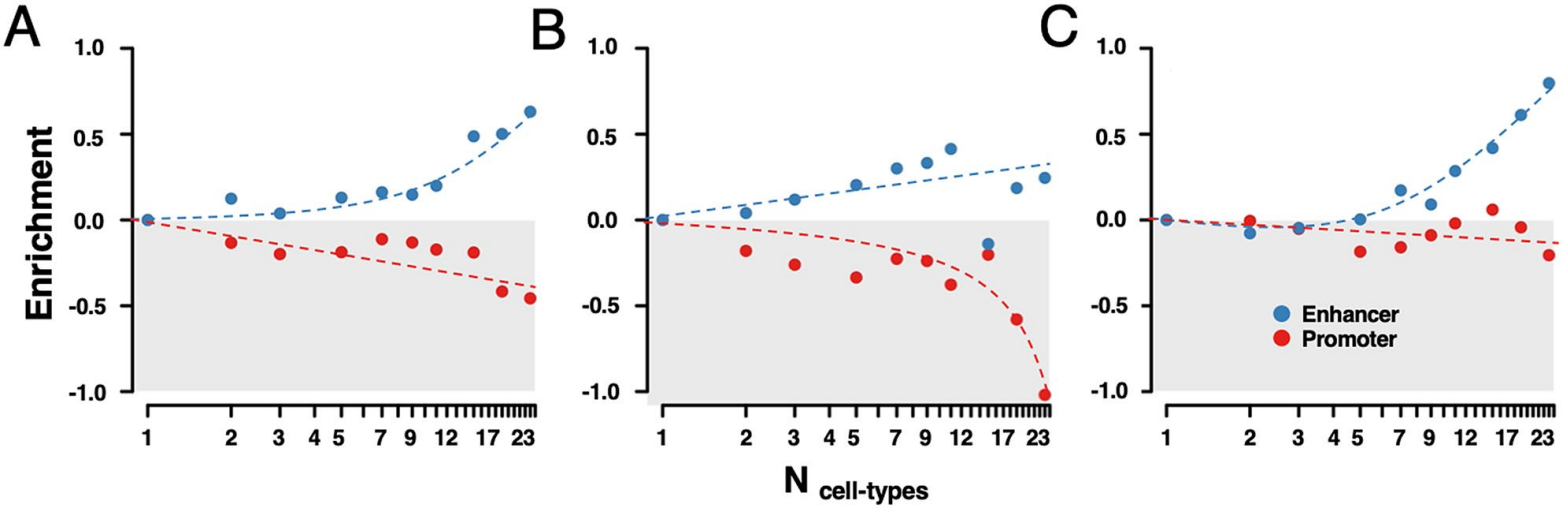
Characteristics of indispensable promoter/enhancer-binding transcription factors (TF) in cell-type specific regulatory networks. Randomly sampling sets of indispensable promoter and enhancer-binding TFs we found that enhancer-binding TFs that were indispensable for an increasing number of cell-type networks were (**A**) enriched with disease-causing genes, (**B**) cancer genes and (**C**) signaling functions. In turn, we found the opposite, when we considered indispensable promoter-binding TFs.

Investigating the role that indispensable promoter/enhancer-binding TFs play in genetic causes of diseases, we considered a set of 3609 genes containing disease-causing mutations^19,20^. We found that such disease genes were enriched with indispensable enhancer-binding TFs in a growing number of cell-type specific regulatory networks (Fig. 4A) while we found the opposite for indispensable promoter-binding TFs. Considering a set of 671 genes causally implicated in oncogenesis as annotated by Sanger Center^21^, we found similar levels of enrichment of cancer genes (Fig. 4B). Furthermore, we utilized 5325 proteins with signaling functions and found that such proteins were enriched with indispensable enhancer-binding TFs in an increasing number of regulatory networks as well (Fig. 4C). Notably, such enrichment levels were not present when we considered tissue networks (Fig. S10). Only cancer genes were enriched as the number of networks increased when we considered generally indispensable TF in cell-type and tissue-specific networks (Fig. S11).

**Figure 5:**
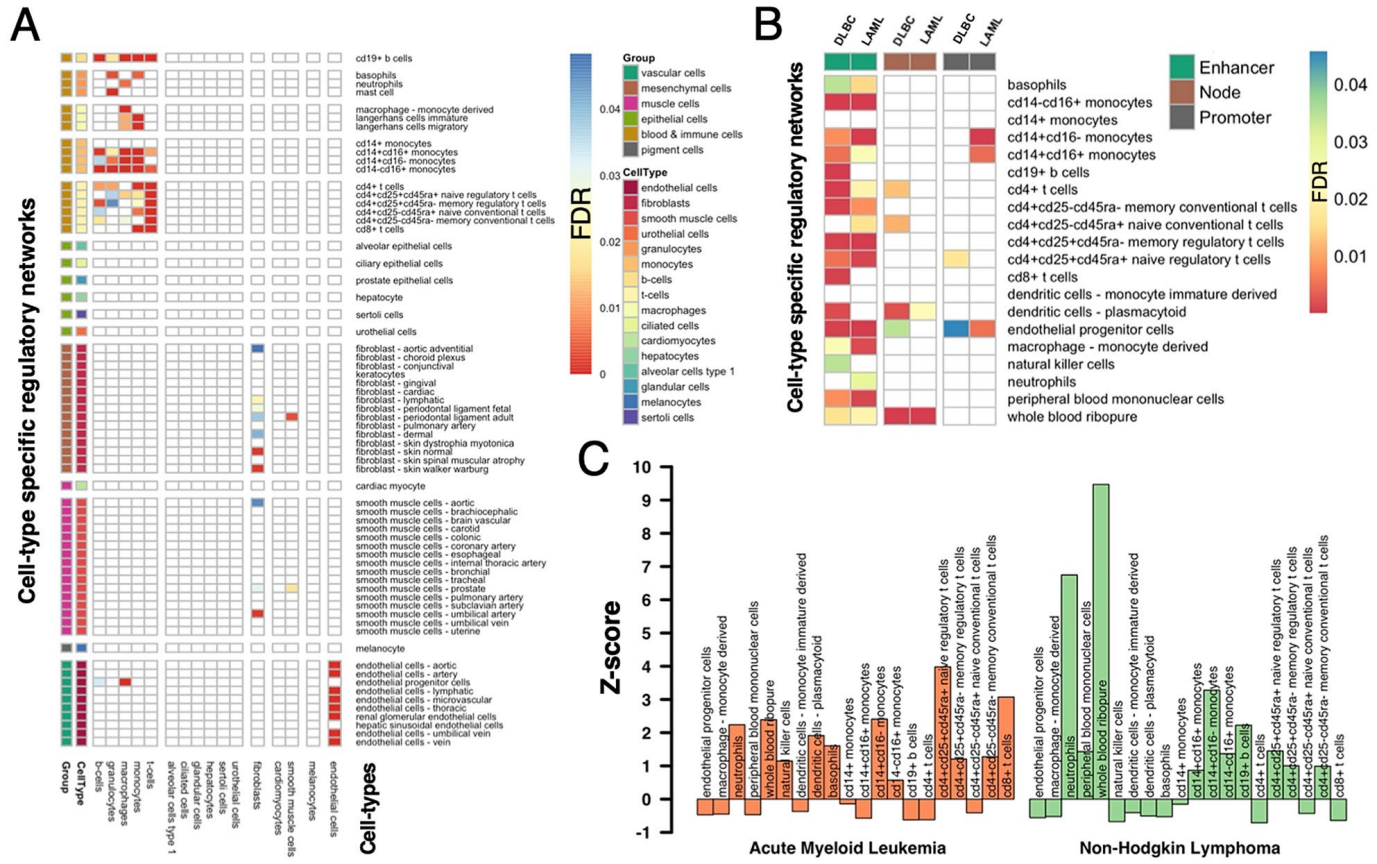
Tissue and cancer specificity of target genes regulated by indispensable enhancer-binding TFs in cell-type specific regulatory networks. In (**A**) we determined the enrichment of cell-type specifically expressed genes in sets of target genes of indispensable enhancer-binding TFs in the corresponding regulatory cell-type network (FDR < 0.05, Fisher’s exact test). We observed that such target genes were enriched with genes expressed in blood and immune-related cell types and endothelial cell types. (**B**) We corroborated our findings by observing the enrichment of genes associated with blood-related cancers such as Acute Myeloid Leukemia (LAML) and Lymphoid Neoplasm Diffuse Large B-cell Lymphoma (DLBC) in sets of target genes of indispensable enhancer-binding TFs in cell-type networks of blood and immune cell. (**C**) Determining the enrichment of gene targets of drugs approved for Acute Myeloid Leukemia and Non-Hodgkin Lymphoma, we found an enrichment in sets of target genes of indispensable enhancer-binding TFs in the cell-type networks of blood and immune cells.

Assuming that target genes of indispensable enhancer-binding TFs are likely cell-type specific, we obtained cell-type specific expressed genes from the Single Cell Type Atlas^14^ where we considered cell-type-specific genes if they had at least four-fold higher mRNA levels in a particular cell type compared to the average level across all other cell types. Specifically, we mapped 66 cell-type networks to 16 different cell types. In Fig. S12, we observed that cell-type specifically expressed genes were indeed enriched in sets of target genes of all enhancer-based TFs in the corresponding cell-type specific regulatory networks. However, our observations were noisy even at a significance level of FDR < 0.05 as enrichment also appeared in irrelevant cell types. Notably, we observed no cell-type specificity when we considered target genes of all promoter-binding TFs (Fig. S12). In the heatmap in Fig. 5A, we found that cell-type specific genes were enriched (P < 0.05, Fisher’s exact test, FDR adjusted) in target genes of indispensable enhancer-binding TFs in blood and immune cell-types and endothelial cells. In turn, target genes of indispensable promoter-binding TFs were enriched in fibroblast and some epithelial cells (Fig. S13). Target genes regulated by generally indispensable TFs were found to be sporadically enriched with cell-type expressed genes, where we could not draw a conclusion about the cell-type specificity (Fig. S14).

Investigating the role of indispensable enhancer/promoter-binding TFs in transitioning between disease and healthy states, we considered 1,982 FDA approved drug targets^16^ and a set of 4808 druggable proteins^18^, indicating that 35% of the cell type networks had druggable genes enriched in target genes of indispensable promoter-binding TFs. In contrast, druggable proteins were found to be significantly enriched in sets of target genes regulated through indispensable promoter-binding TFs in 51% of the cell-type networks. Furthermore, drug targets and different families of druggable genes were enriched in less than 5% of all the cell-type networks in target genes of promoter-, enhancer-binding, or generally indispensable transcription factors (Fig. S15). Focusing on blood and immune cell-types, we obtained a list of genes associated with immune disorders from the Genetic Association Database^22^. Focusing on immune-related disease, we considered the top six diseases with the highest number of associated genes. Specifically, we obtained 763 genes in asthma, 453 in celiac disease, 583 in diabetes type-1, 633 in lupus, 676 in multiple sclerosis, and 585 in rheumatoid arthritis. We observed that those genes were significantly enriched in sets of target genes of indispensable enhancer-binding TFs in blood and immune cell networks. In turn, we did not find enrichments of disease-associated genes in target genes regulated through promoter-binding and generally indispensable TFs (Fig. S16). Furthermore, we also obtained a list of genes that are overexpressed in Acute Myeloid Leukemia (LAML) and Lymphoid Neoplasm Diffuse Large B-cell Lymphoma (DLBC)^15^. In particular, we found that blood-related cancer-specific genes were enriched in sets of target genes of indispensable enhancer-binding TFs in the corresponding cell-type networks. In turn, we did not observe such enrichment when we considered target genes regulated through promoter-binding or generally indispensable TFs (Fig. 5B). As a corollary, we hypothesized that target genes regulated through enhancer-binding TFs might also be targeted by drugs approved for blood-related cancers. Focusing on FDA-approved drugs and their targets from DGIdb^18^, we found that genes targeted by approved drugs for Acute Myeloid Leukemia and Non-Hodgkin Lymphoma were enriched among target genes of indispensable enhancer-binding TFs (Fig. 5C). In turn, we did not observe such enrichment when we considered target genes regulated through promoter-binding or generally indispensable TFs (Fig. S17).

## Discussion

In this work, we classified regulatory edges as promoter or enhancer regulated in 257 separate regulatory networks, corroborating previous observations, suggesting that enhancer-regulated edges tend to be tissue specific^23^. We then determined TFs that were indispensable for topological control in different tissue and cell-type specific regulatory networks through promoters and enhancer-binding regulatory links. Utilizing structural controllability of linear dynamics we obtained sets of driver nodes^13^ in tissue and cell-type specific regulatory network, and assessed the role of promoter/enhancer binding of each TF for the controllability of the underlying network through removing the corresponding promoter/enhancer-binding edges and recalculating the driver node-set. If the number of driver nodes increased, we considered the underlying TF indispensable for control of the underlying network through promoter/enhancer regulation as more driver nodes need to be tweaked to achieve structural controllability compared to the unperturbed network. If the driver node set’s cardinality did not change or decreased, we did not consider such a TF as relevant for control. Such an approach is different from previous methods that assessed the topological impact on the controllability of a network as a function of single edges^13^ or approaches where nodes are considered to control each link independently^24^. In fact, our approach allows us to investigate the impact that TFs have through regulatory elements such as enhancers and promoters on the controllability of the underlying regulatory network. As a benchmark, we also determined generally indispensable^10^ TFs through their impact on the cardinality of driver nodes after deleting a given TF as a whole irrespectively of the binding nature of regulatory elements, allowing us to compare the differences in influence of regulatory elements on structural controllability of regulatory networks.

Our analysis revealed that the distribution of the fraction of promoter and enhancer edges between tissue networks and cell type networks were significantly different. In particular, we found that cell-type networks had a larger fraction of enhancer edges compared to tissue regulatory networks. Such cellular differences are usually masked at the tissue level as multiple cell types are averaged, which is problematic when regulatory properties of interest are limited to a subpopulation of cells within a given tissue^25^. Furthermore, gene regulatory programs in different cell-type are also influenced by epigenetic factors. Such characteristics suggest that cell-type specific control may not be fully defined by either promoter or enhancer controlled regulation^26,27^, blurring their influence and presence in regulatory networks of different cell types.

We found that the cumulative frequency distribution of the number of networks controlled by a given TF through promoter (or enhancer) binding decayed exponentially, indicating that a minority of such TFs appeared indispensable for the control of a large number of networks and vice versa. However, the distribution of indispensable enhancer-binding TFs decayed faster in tissue-specific networks, further indicating the higher relevance of promoter-binding regulatory links in tissue networks. In turn, the decay in the corresponding distribution in cell-type specific was similar, suggesting that enhancer-binding plays a more prominent role in cell-type specific regulatory interactions.

Since enhancer-binding edges tend to appear in a small number of tissue networks, we observed that target genes of enhancer-binding TFs were tissue-specific, corroborating previous results^23,28^. In turn, target genes regulated through promoter-binding links were not observed to be tissue-specific. As a result, we expected target genes of indispensable enhancer-binding TFs to be tissue-specific as well. To our surprise, we observed the complete opposite as target genes of indispensable promoter-binding TFs were tissue-specific in 30 out of 31 tissues. The observed discrepancy may be rooted in the observation that the target genes of TFs that bind promoters are ubiquitous, while target genes of enhancer-binding TFs are more granular, overlapping with targets of promoter-binding TFs. This disposition is completely dissolved, when we consider target genes of indispensable promoter and enhancer-binding TFs, as they constitute a small fraction of target genes of all TFs. Such a result suggests that topological control through promoter-binding edges is partially responsible for the biological governance of cellular functions at the tissue level. We further corroborated this hypothesis by showing cancer-specific genes to be enriched in sets of target genes of indispensable promoter-binding TFs in 16 different cancer types in the corresponding tissue networks while we hardly found any enrichment when we considered enhancer-binding or generally indispensable TFs.

Investigating target genes further, we found that 80% of all the tissue-specific networks had druggable proteins enriched in target genes of indispensable promoter-binding TFs. In more detail, we found ion channels, G protein-couple receptors, and drug targets to be enriched in at least 20% of the tissue networks. Such results indicated that target genes that were regulated by indispensable promoter-binding TFs are involved in signaling pathways and could be modulated using small molecule compounds. Hence, we expected the targets of approved cancer drugs to be enriched in sets of target genes of indispensable promoter-binding TFs. Indeed, corresponding target genes of such TFs were enriched with drug targets approved for cancer in the corresponding tissue, suggesting that indispensable promoter-binding TFs potentially can modify signaling to obtain a desirable therapeutic effect through their target genes. As a corollary, we also found that the targets of six selected drugs were enriched in target genes of indispensable promoter-binding TFs in the corresponding tissue regulatory networks, reinforcing our findings. Comparing our results to generally indispensable TFs, we found that controllability achieved through promoter edges defines tissue specificity much better than nodal dynamics alone.

As frequency distribution of the networks controlled by indispensable promoter and enhancer-binding TFs are similar in cell-type networks, we investigated biological characteristics of such TF as a function of their appearance in an increasing number of cell-type specific regulatory networks. In particular, we found that cancer causal genes were enriched with indispensable enhancer-based TFs as they control an increasing number of cell types. Similarly, disease genes that carry causal mutations and signaling processes involved in pathways whose abnormal activation could lead to disease were found to be enriched as well. In turn, promoter-binding and generally indispensable TFs did not show any such characteristics, suggesting that the topological placement of indispensable enhancer-binding TFs have signaling relevance to disseminate biological information in a large number of cell types, whose dysregulation may have a detrimental effect. In the absence of such enrichment signals when we considered TFs in tissue regulatory networks, we surmise that the difference in cell type and tissue networks could be explained by the increase in granularity of cellular components in cell-type networks.

Following tissue network results, we found that target genes regulated through enhancer-binding TFs were cell-type specific in corresponding networks corroborating previous results^23,28^ while target genes of promoter-binding TFs were not cell-type specific. Investigating target genes regulated through indispensable TFs, we expected that target genes of indispensable promoter-binding TFs could be cell-type specific. While epithelial cells and fibroblasts specific genes were mildly enriched in the sets of target genes of indispensable promoter-binding TFs, we instead observed that target genes of indispensable enhancer-binding TFs in blood and immune-related and endothelial cells were strongly cell-type specific. Also, our method identified a small number of TFs that achieved cell-type specificity through its regulatory mechanism. We corroborate such a hypothesis by showing that the genes associated with six immune diseases and blood-related cancers are also enriched in sets of target genes of indispensable enhancer-binding TFs. Assuming that disruption of the regulation of target genes by indispensable enhancer-binding TFs can cause diseases, we hypothesized that these TFs also regulate drug targets associated with blood cancer. Although none of the cell-type networks were significantly enriched with druggable genes and drug targets, we did find that targets of the drugs approved for Acute Myeloid Leukemia and Non-Hodgkin Lymphoma were enriched among target genes regulated by indispensable enhancer-binding TFs in blood-related cell-type networks. Since it is critical for suitable drug targets to limit potential side effects, a regulatory network-specific druggable genome that plays a significant role in the control process identified by our method may serve as an important starting point for therapeutic research.

## Methods

### Tissue and cell type specific regulatory networks

We obtained 111 tissue and 146 cell type specific transcriptional regulatory networks from^12^. In particular, such networks were inferred using expression profiles of Cap Analysis of Gene Expression (CAGE) analyses from the FANTOM5 project^29,30^ through genome-wide mapping of promoters and enhancers, linking transcription factors to binding promoters and enhancers that were linked to target genes. CAGE maps regions of transcription initiation with high resolution and sensitivity, allowing the identification of active promoters and enhancers. Weighted, tissue-specific links between transcription factors and regulatory elements (*i.e.* enhancers and promoters) were inferred using transcription factor binding motifs and tissue-specific expression of target elements. Furthermore, interactions between regulatory elements and target genes were inferred based on genomic distance and joint expression in the given tissue. To construct tissue and cell-type specific regulatory networks between transcription factors and target genes, we utilized enhancer-gene and promoter-gene annotations from^12^ that were expressed in the corresponding tissue/cell type and mapped transcription factors with binding motifs to the corresponding promoters/enhancers, allowing us to classify each regulatory link between a transcription factor and target gene as enhancer and/or promoter regulated.

### Controllability analysis

We identified a minimum set of driver nodes in each of the regulatory circuits that are defined as sufficient to ensure the structural controllability of linear dynamics^13^. Such a structural controllability problem can be mapped to a maximum matching problem that can be solved in polynomial time using the Hopcroft-Karp algorithm^31^. Specifically, we mapped directed links between transcription factors and target genes to edges between partitions of nodes that start and end edges. In the matching, a subset of edges *M* is a matching of maximum cardinality in a directed network if no two edges in *M* share a common starting and ending vertex. Vertices that do not appear in *M* are unmatched and are nodes that structurally control the underlying network^13^. As a corollary, a maximum matching implies the presence of a minimum set of such driver nodes of size *N*_*D*_.

#### Edge-based controllability

To assess the impact of regulatory elements such as enhancers and promoters on the controllability of the underlying directed network we applied the following heuristic: After removing all regulatory interactions that involve a transcription factor that binds either a promoter (P) or enhancer (E) we determined the size of the set of driver nodes in the changed network, ${N}_{D}^{P,E}$. If ${N}_{D}^{P,E}>{N}_{D}$ the promoter/enhancer-binding transcription factor was classified as indispensable for the control of the underlying network as the number of control nodes increased in the changed network. If ${N}_{D}^{P,E}\le {N}_{D}$, the promoter/enhancer-binding transcription factor is irrelevant for control as the number of driver nodes decreased or remained unchanged.

#### Node-based controllability

To classify a TF as generally indispensable, we removed the whole TF and its interactions from the network and determined the size of the driver nodes of the network thus obtained, ${N}_{D}^{^{\prime}}$. If ${{N}_{D}^{^{\prime}}>N}_{D}$, we considered the TF to be generally indispensable for the control of the underlying network^10^.

### Enrichment analysis

#### Enrichment scores

The enrichment of TFs with a certain characteristic *A* was determined as a function of the number of regulatory networks *k*, that a given TF was indispensable for. In particular, ${N}_{\ge k}^{A}$ is the number of TFs with characteristic *A* that were found indispensable in $\ge k$ regulatory networks. Randomly sampling a set of TFs with characteristic *A*, we calculated the corresponding random number, ${N}_{\ge k}^{r,A}$. The enrichment of these TFs with characteristic *A* in at least *k* regulatory networks was then defined as ${E}_{\ge k}^{A}=l{g}_{2}\left(\frac{{N}_{\ge k}^{A}}{{N}_{\ge k}^{r,A}}\right)$. In this case, ${E}_{\ge k}^{A}>0$ points to an enrichment of feature *A* and vice versa. In particular, TFs with feature *A* were randomly sampled 10,000 times and averaged enrichment values thus obtained.

#### Z-values

Furthermore, in a group *i* of proteins the corresponding number of proteins with a certain characteristic *A*, ${N}_{i}^{A}$ (*e.g.* a drug target) were determined. Randomly sampling a set of proteins with characteristic *A*, we calculated the corresponding average random number ${\mu }_{i}^{r,A}$ and standard deviation ${\sigma }_{i}^{r,A}$ of control proteins with *A*. Finally, we defined the enrichment of proteins with characteristic *A* in a group *i* of proteins as their Z-score, ${Z}_{i}^{A}=\frac{{N}_{i}^{A}-{\mu }_{i}^{r,A}}{{\sigma }_{i}^{r,A}}$.

### Data sources

#### Disease genes

We collected 4,015 genes that were considered causal for a disease from the human phenotype ontology database (HPO)^19^, that is based on the Online Mendelian Inheritance in Man (OMIM) database^20^. For cancer-related genes, we used a representative set of 723 cancer genes annotated by the Sanger Center as causally implicated in oncogenesis^21^. We also obtained genes that are implicated in six immune-related disorders from the Genetic Association Database (GAD)^22^. Specifically, we obtained 763 genes associated with asthma, 453 with celiac disease, 583 with diabetes type 1, 633 with lupus, 676 with multiple sclerosis, and 585 with rheumatoid arthritis. Furthermore, we used 6,860 human signaling proteins from Gene Ontology utilizing term GO:0023052^32^.

#### Drug targets and druggable genes

For genes associated with the transition between disease and healthy states, we also obtained a set of 2,166 drug targets that were approved by the Food and Drug Administration (FDA) from the DrugBank database^16^. Furthermore, we accounted for 5,776 genes that were annotated as druggable since these proteins carried domains that were suitable to interact with drugs^17^. Druggable genes were further categorized into gene families, including G protein-coupled receptor (798) and ion channels (316) from DGIdb^18^. We also collected 85,832 interactions between drugs and their drug targets from the same source.

#### Tissue specific and cancer genes

As for tissue and cancer specificity, we collected a list of tissue enriched, tissue enhanced, and group enhanced genes from The Human Protein Atlas Database^14^, capturing 37 different tissues. We considered a gene tissue-specific if its mRNA levels in a particular tissue are at least four-fold higher than the corresponding average expression level in all other tissues, which is the union of tissue enriched, tissue enhanced, and group enhanced genes. We also obtained a list of cancer-specific genes, which is the union of cancer-enriched, cancer-enhanced, and group enhanced genes, capturing 18 different types of human cancer from The Pathology Atlas Database^15^. The cancer-specific genes for AML and DLBC were obtained from the Supplementary Table 2 of^15^. Numbers of tissue and cancer specific genes of each tissue and cancer are listed in Supplementary Table 1.

#### Cancer drugs

We obtained a list of drugs approved for 18 different cancer types from the National Cancer Institute (https://www.cancer.gov/about-cancer/treatment/drugs/cancer-type). We also obtained a list of genes that interact with those cancer drugs based on the National Cancer Institute Cancer Gene Index from DGIdb^18^, which curates drug gene interaction from various sources. In Supplementary Table 2, we list the number of approved drugs as well as the total number of unique targets for those drugs in each cancer type. In the number of targets column of Supplementary Table 2, we only accounted for targets that were present in at least one of our tissue-specific regulatory networks.

#### Cell-type specific genes

We obtained cell-type-specific genes from the Single Cell Type Atlas^14^ database, capturing 51 different cell types. We considered genes cell-type-specific if they had at least four-fold higher mRNA levels in a particular cell type compared to the average level over all other cell types. Specifically, we considered genes classified as cell type enriched, group enriched, and cell type enhanced genes by the source database^14^ to be cell-type-specific. We mapped 66 cell-type networks to 16 different cell types that were further categorized into six different groups based on the source database^14^. In Supplementary Table 3, we list the cell types, their grouping, and the number of cell-type-specific genes for the cell types that we used in our analysis.

## Supplementary Information

Supplementary Information.
