# Supplementary material for: Promoter/enhancer-based controllability of regulatory networks
Source: Sci Rep. 2022 Mar 3;12:3528. doi: 10.1038/s41598-022-07035-4 (PMC8894475; doi:10.1038/s41598-022-07035-4)
Supplement: Supplementary file 1 — Supplementary Information. [file 41598_2022_7035_MOESM1_ESM.pdf]

Promoter/Enhancer-based Controllability of Regulatory Networks – Suppl. Materials

Prajwal Devkota[1,#], and Stefan Wuchty[1,2,3,*]

[1] Department of Computer Science, University of Miami, FL 33146, USA
[2] Department of Biology, University of Miami, FL 33146, USA
[3] Sylvester Comprehensive Cancer Center, Univ. of Miami, Miami, FL 33136, USA
[#] current address: Scipher Medicine Inc., Waltham, MA 02453, USA
* wuchtys@cs.miami.edu

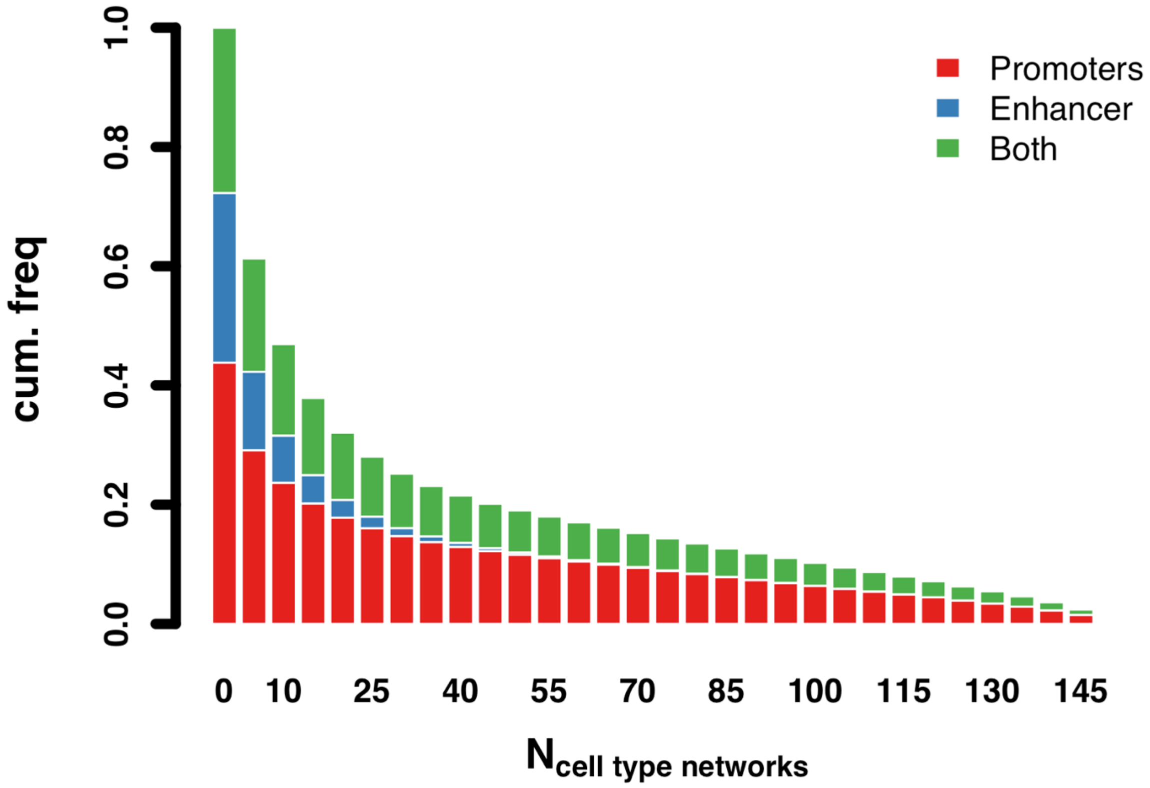

Figure S1. Counting the appearance of regulatory links in different cell-type specific networks, we observed that enhancer-binding regulatory interactions were more likely to be cell-type specific while promoter regulated edges tend to appear more frequently in many different cell-types.

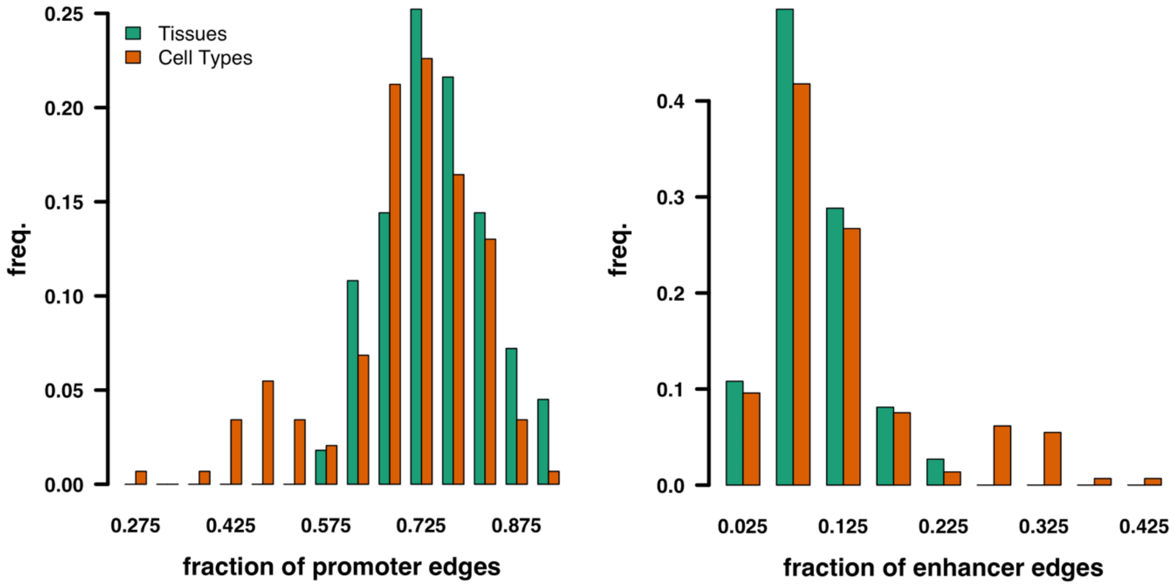

Figure S2. Counting the number of promoter and enhancer-binding regulatory interactions, we observed that regulatory networks in tissues are significantly more populated with promoter-binding interactions, while we observe the opposite, considering enhancer-binding interactions in cell-type specific networks (Wilcoxon rank-sum test, P < 0.05).

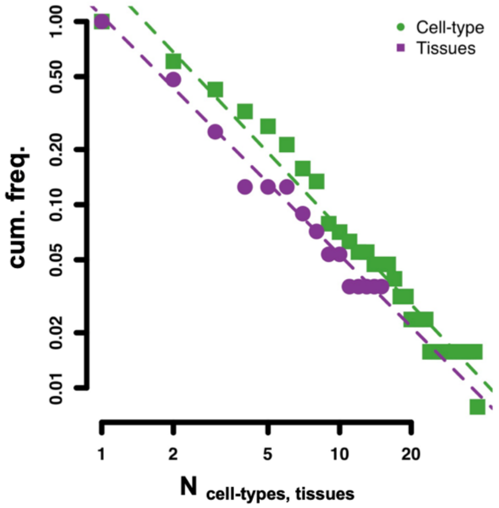

Figure S3. We observed that the cumulative frequency distribution of generally indispensable TF followed power-law distribution in both, tissue-specific networks and cell-type specific regulatory networks.

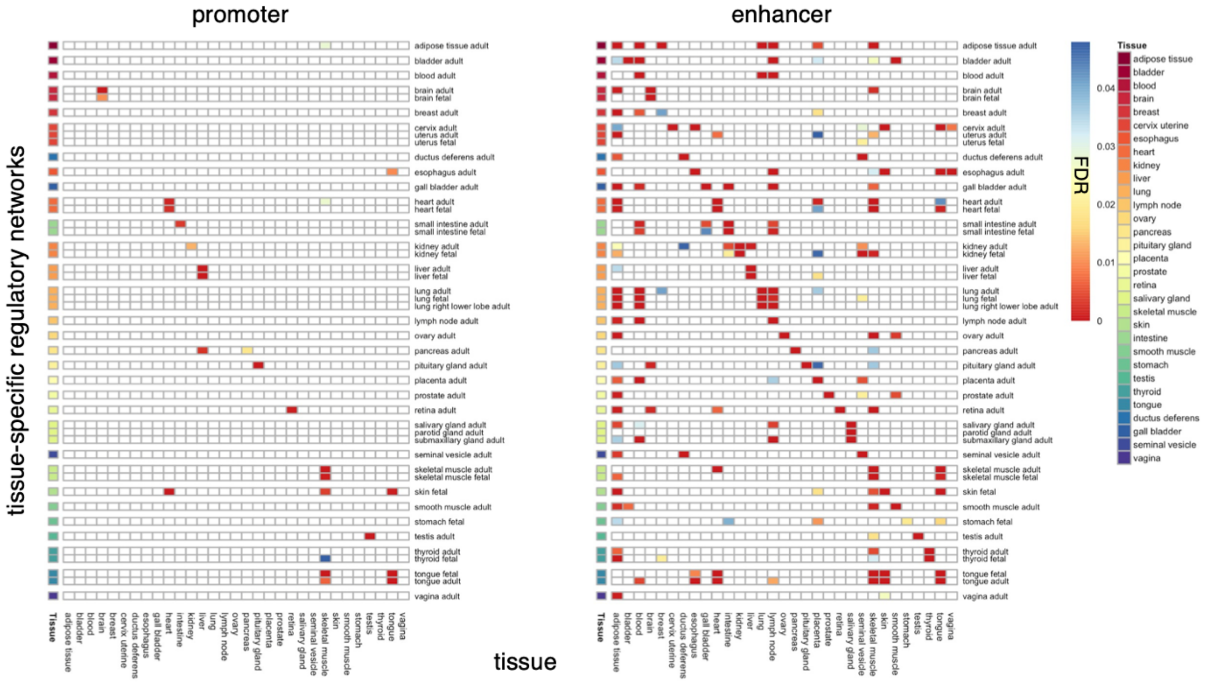

Figure S4. In the heatmaps, we indicated the enrichment of sets of target genes regulated through promoters and enhancers in tissue-specific regulatory networks with expressed genes in 31 different corresponding tissues (FDR < 0.05, Fisher's exact test). We observed that target genes regulated through enhancers were enriched in almost all tissues, while we only found weak enrichment of genes that were regulated through promoters.

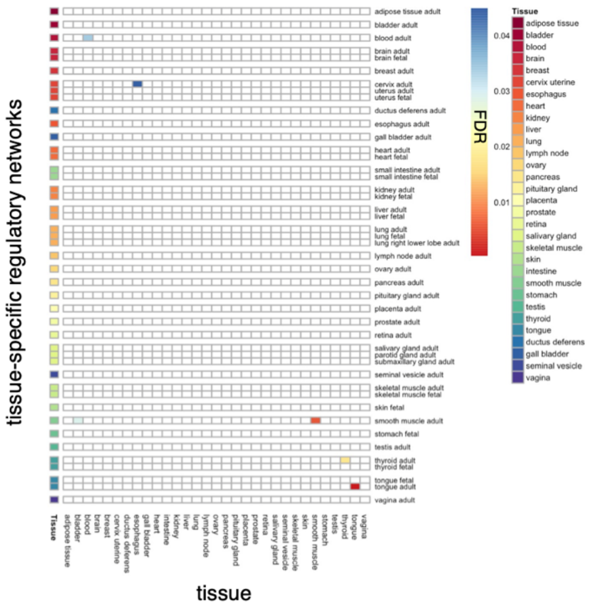

Figure S5. Enrichment of sets of target genes regulated through indispensable enhancer-binding transcription factors in tissue-specific regulatory networks with tissue-specific expressed genes (FDR < 0.05, Fisher's exact test). We observed the target genes were hardly enriched in most of the tissues.

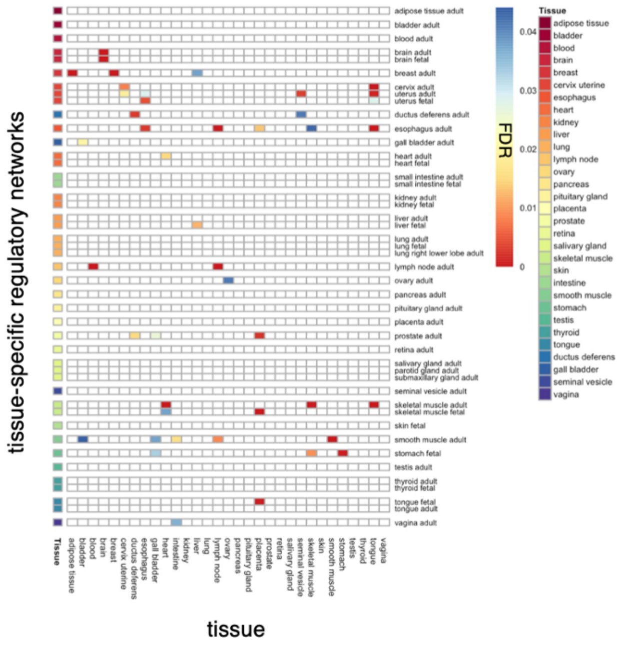

Figure S6. Enrichment of sets of target genes regulated through generally indispensable transcription factors in tissue-specific regulatory networks with tissue-specific expressed genes (FDR < 0.05, Fisher's exact test). We observed the target genes were hardly enriched in most of the tissues.

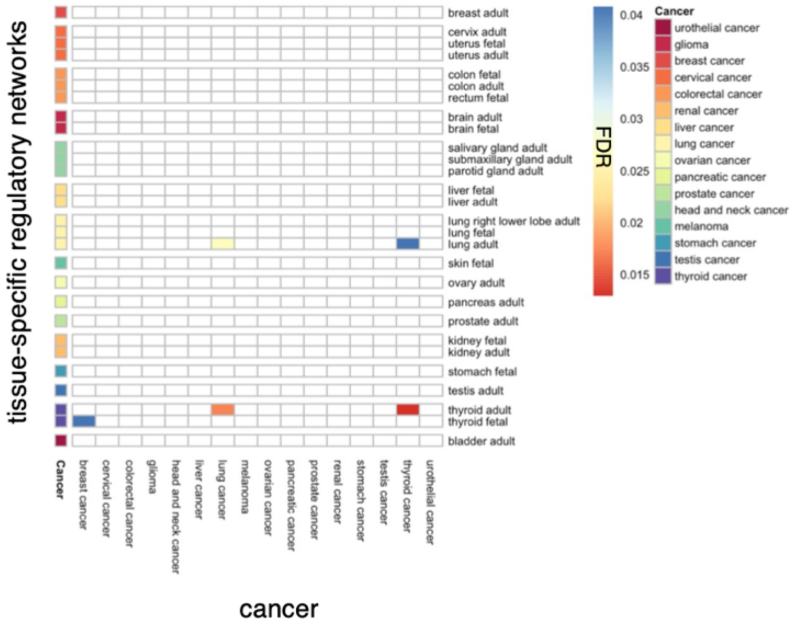

Figure S7. Considering 16 different cancer-types we determined the enrichment of target genes regulated through indispensable enhancer-binding transcription factors in tissue-specific regulatory networks with such cancer-specific expressed genes (FDR < 0.05, Fisher's exact test). We observed that the target genes were hardly enriched in most of the cancer types.

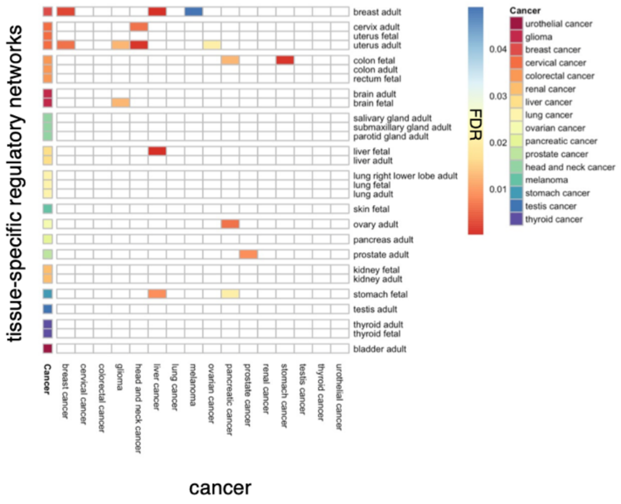

Figure S8. Considering 16 different cancer-types we determined the enrichment of target genes of generally indispensable transcription factors in tissue-specific regulatory networks with cancer-specific expressed genes in sets (FDR < 0.05, Fisher's exact test). We observed that the target genes were hardly enriched in most of the cancer types.

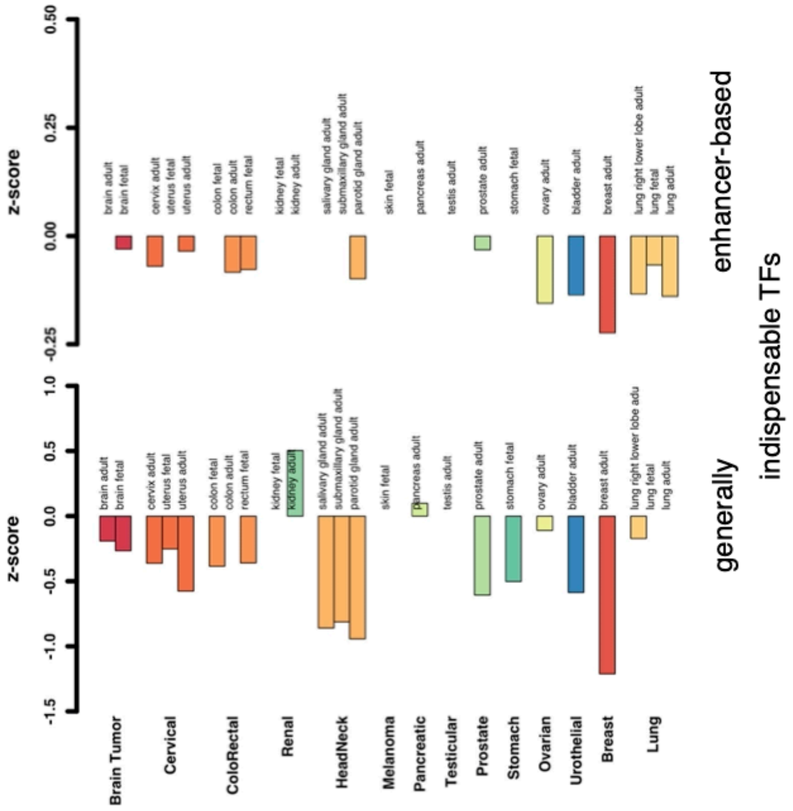

Figure S9. Considering gene targets of drugs approved for different cancers we observed that target sets of enhancer-binding and generally indispensable TFs in corresponding tissue-specific regulatory networks were diluted with drug targets.

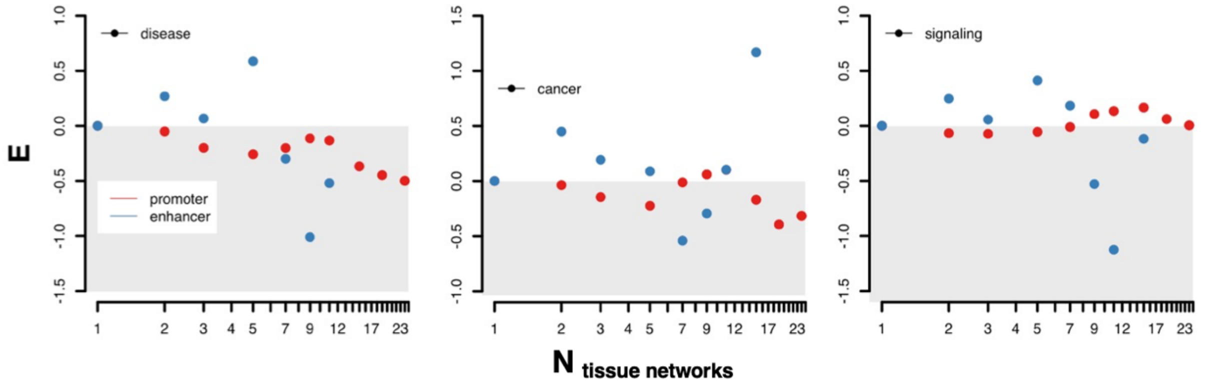

Figure S10. TFs that were indispensable for the control of a growing number of tissue networks through promoters and enhancer-binding were not increasingly enriched for disease, cancer, and signaling genes.

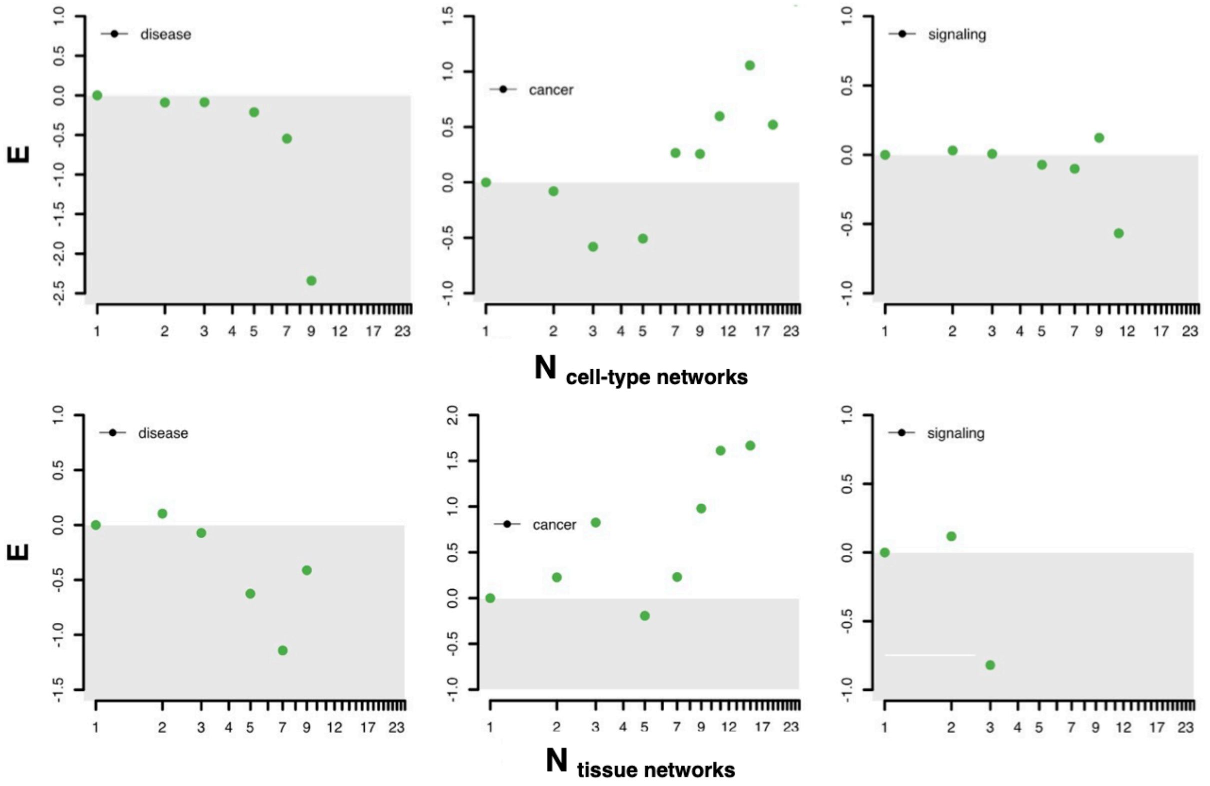

Figure S11. TFs that generally were indispensable for a growing number of *cell-type* (upper panel) and *tissue* (lower panel) specific networks were increasingly enriched with cancer genes compared to disease and signaling genes.

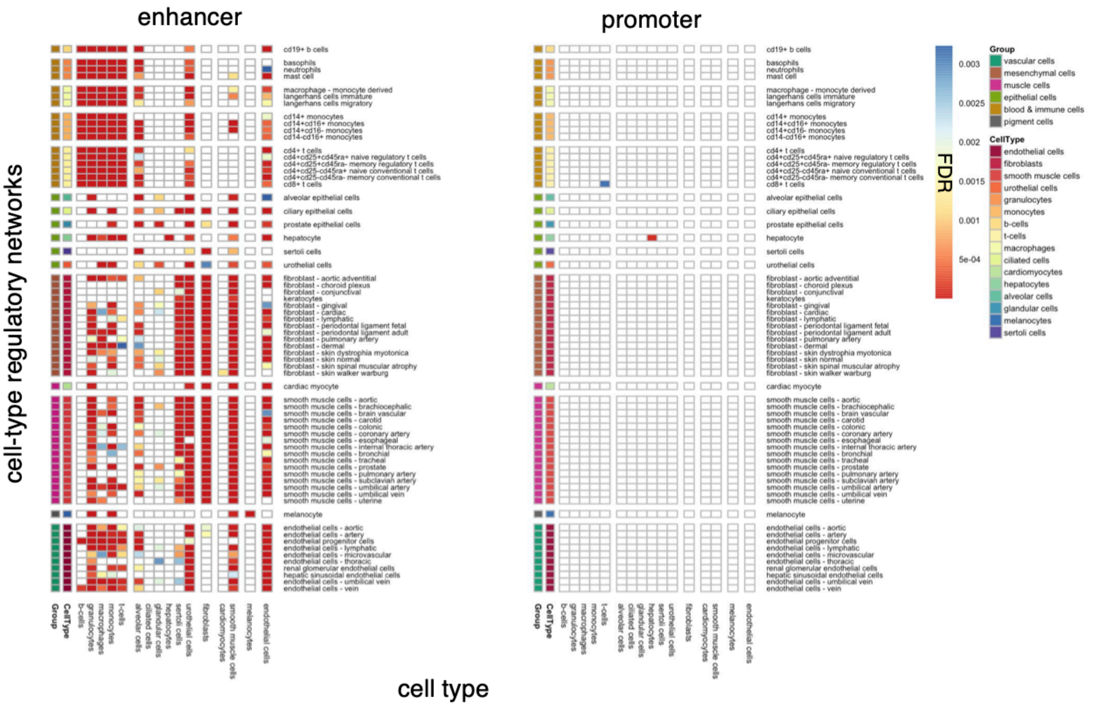

Figure S12. In the heatmaps, we indicated the enrichment of cell-type-specific expressed genes in sets of target genes of enhancer- and promoter-binding transcription networks in cell-type specific regulatory networks (FDR < 0.05, Fisher's exact test).

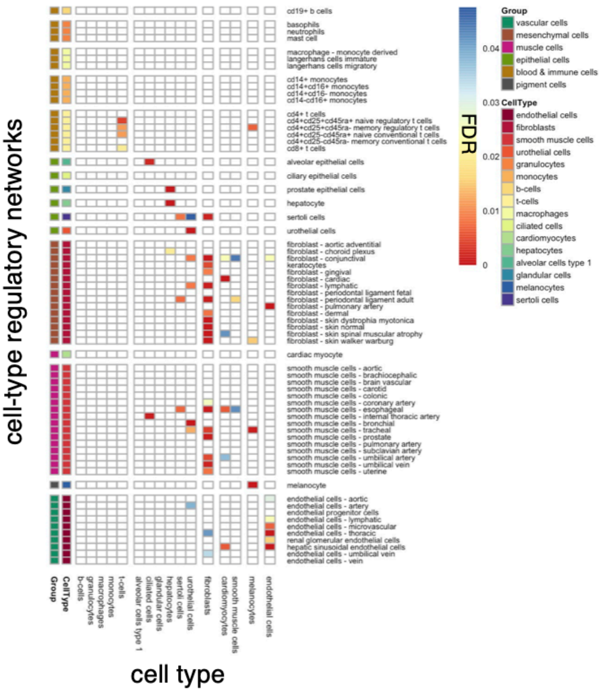

Figure S13. Obtaining a list of cell-type specific expressed genes we indicate their enrichment in sets of target genes controlled through indispensable promoter-binding TFs in the corresponding cell-type network (FDR < 0.05, Fisher's exact test). We observed that target genes of indispensable promoter-binding TFs were mildly enriched in epithelial cells and fibroblasts cell types.

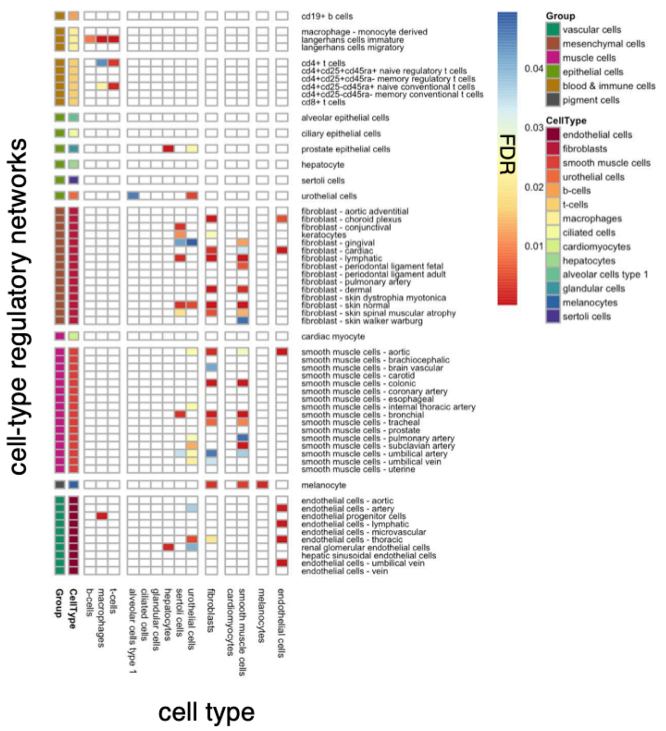

Figure S14. Obtaining a list of cell-type specific expressed genes we indicate their enrichment in sets of target genes controlled through generally indispensable TF in the corresponding cell-type network (FDR < 0.05, Fisher's exact test). We observed that target genes of generally indispensable TFs were mildly enriched in epithelial and smooth muscle cells.

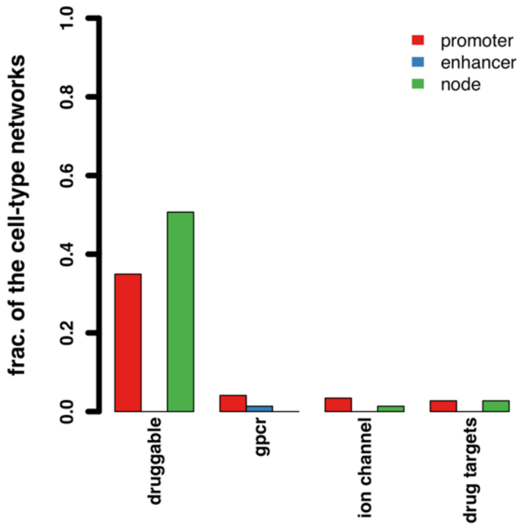

Figure S15. Enrichment of drug-relevant genes in sets of target genes regulated by indispensable promoter-binding TFs, indispensable enhancer-binding TFs, and generally indispensable TFs in cell-type specific regulatory networks.

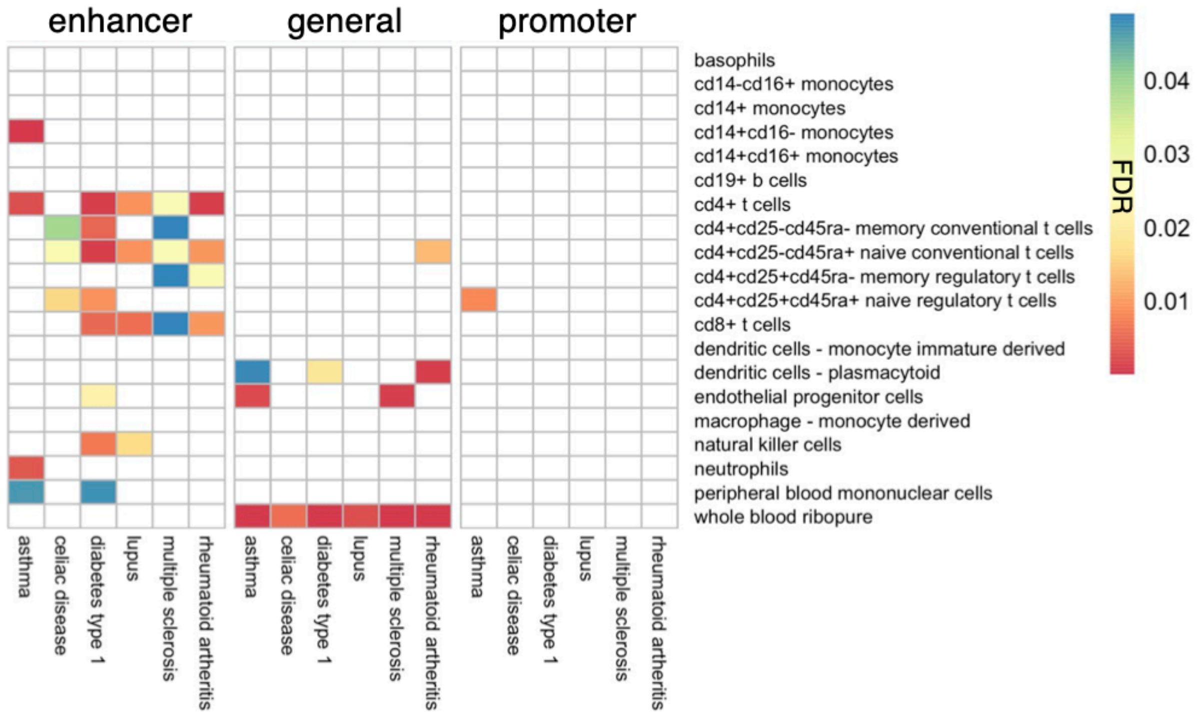

Figure S16. Enrichment of immune disease genes in target genes regulated by promoter/enhancer-binding and generally indispensable TFs in blood and immune cell-type networks.

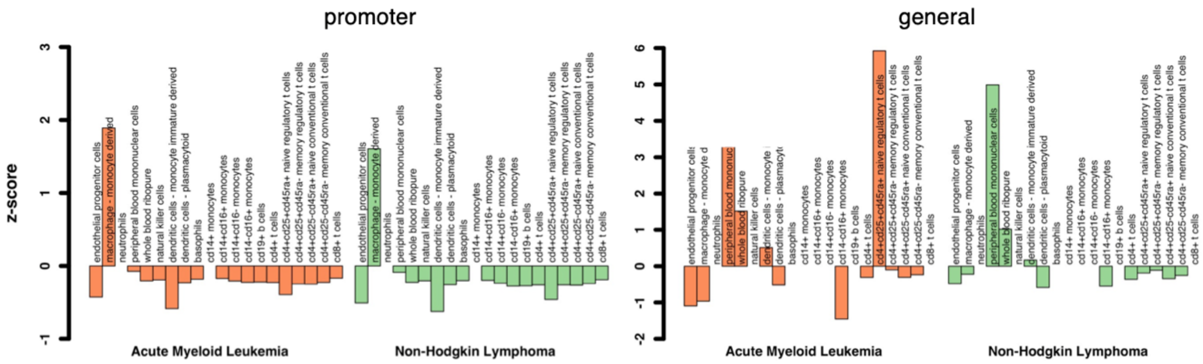

Figure S17. Enrichment of gene targets of drugs that treat Acute Myeloid Leukemia and Non-Hodgkin Lymphoma in sets of target genes regulated by promoter-binding and generally indispensable TFs in blood related cell-type networks.

Tissue name	Tissue specific genes	Cancer in tissue	Cancer specific genes
adipose tissue	164		
bladder	67	urothelial cancer	111
blood	952	AML	704
blood		DLBC	272
brain	2101	glioma	613
breast	128	breast cancer	128
cervix uterine	102	cervical cancer	101
ductus deferens	104		
esophagus	255		
gall bladder	144		
heart	324		
intestine	634		
kidney	339	renal cancer	248
liver	810	liver cancer	425
lung	200	lung cancer	89
lymph node	1204		
ovary	135	ovarian cancer	119
pancreas	311	pancreatic cancer	174
pituitary gland	271		
placenta	415		
prostate	84	prostate cancer	202
retina	279		
salivary gland	246	head and neck cancer	239
seminal vesicle	143		
skeletal muscle	749		
skin	356	melanoma	223
smooth muscle	95		
stomach	112	stomach cancer	127
testis	1354	testis cancer	371
thyroid	149	thyroid cancer	160
tongue	343		
vagina	81		
colon, rectum		colorectal cancer	153

Table S1. Number of genes that were significantly expressed in tissues and corresponding cancer types.

Cancer	# of Drugs	# of Targets
Brain Tumor	2	7
Breast	16	75
Cervical	3	13
ColoRectal	7	20
HeadNeck	4	31
Liver	1	2
Lung	8	69
Melanoma	2	4
Ovarian	7	103
Pancreatic	4	27
Prostate	5	24
Renal	2	4
Stomach	5	18
Testicular	4	81
Thyroid	1	1
Urothelial	3	50

Table S2. Number of approved drugs and corresponding gene targets for 16 cancer types.

Group	Celltype	Celltype specific genes
Blood & immune cells	granulocytes	420
Blood & immune cells	monocytes	481
Blood & immune cells	b-cells	243
Blood & immune cells	t-cells	361
Blood & immune cells	macrophages	378
Epithelial cells	urothelial cells	406
Epithelial cells	ciliated cells	69
Epithelial cells	hepatocytes	810
Epithelial cells	alveolar cells	665
Epithelial cells	glandular cells	213
Epithelial cells	sertoli cells	463
Mesenchymal cells	fibroblasts	232
Muscle cells	smooth muscle cells	328
Muscle cells	cardiomyocytes	743
Pigment cells	melanocytes	355
Vascular cells	endothelial cells	240

Table S3. Number of significantly expressed genes in given cell types.
